# Gut microbiota and physical activity level: characterization from sedentary to soccer players

**DOI:** 10.5114/biolsport.2024.134759

**Published:** 2024-01-30

**Authors:** Cristian Petri, Gabriele Mascherini, Pascal Izzicupo, Diletta Rosati, Matteo Cerboneschi, Serena Smeazzetto, Luis Suarez Arrones

**Affiliations:** 1 Department of Sports and Computer Science, Section of Physical Education and Sports, Universidad Pablo de Olavide, Seville, Spain; 2 Department of Experimental and Clinical Medicine, University of Florence, 50134 Florence, Italy; 3 Department of Medicine and Aging Sciences, University “G. D’Annunzio” of Chieti-Pescara, Via L. Polacchi, 11, 66100 Chieti, Italy; 4 Department of Medical Biotechnologies, University of Siena, 53100 Siena, Italy; 5 NEXT Genomics Srl, Sesto Fiorentino, 50019 Florence, Italy

**Keywords:** Microbiome, Athletes, Sport, Health, Training

## Abstract

Evidence of the relationship between physical activity and gut microbiota composition is steadily increasing. The purpose of the study is to compare the gut microbiota composition of a group of elite male soccer players with a group of subjects with different physical activity levels. Cross-sectional studies were performed on 91 healthy young males, in detail: 17 elite soccer players (23.7 ± 4.2 yrs, BMI 23.2 ± 1.2 kg/m^2^); 14 with high levels of physical training (24.5 ± 5.6 yrs, BMI 22.7 ± 0.8 kg/m^2^); 23 with moderate levels of physical training (29.3 ± 3.9 yrs, BMI 22.5 ± 0.8 kg/m^2^); and 37 healthy men without exercise habits (28.1 ± 5.9 yrs, BMI 22.4 ± 1.0 kg/m^2^). Relative microbiota composition was determined by analyzing DNA extracted from stool samples. The quality and quantity of extracted DNA were assessed using a Qubit Fluorometer. Differences between subjects’ populations were analyzed using a one-way ANOVA, and Bonferroni’s post-hoc test was employed to identify localized effects. Elite soccer players and subjects with high physical activity levels showed a significantly higher prevalence of the nine microbiota populations analyzed than subjects with moderate physical training or who were sedentary. No differences were found in the Firmicutes to Bacteroidetes ratio among the different study populations. This study reports the gut microbiota parameters of elite footballers for the first time. In addition, it brings new insights into the effects of different levels of physical activity on the composition of the gut microbiota.

## INTRODUCTION

The gut microbiota is a broad term for the billions of microorganisms within the gastrointestinal tract, including bacteria, fungi, and viruses [1]. Investigation of the gut microbiota is a field of research that is attracting growing interest because of its potential role in health and disease [2]. These microorganisms inhabit the human intestine, forming a complex community that interacts within itself and with the host; however, its composition depends on the host’s genetics and numerous environmental factors [3], and the latter plays a dominant role in its continuous modification.

Among the environmental factors that have been determined to have a role in modifying the gut microbiota are lifestyle, in terms of eating habits and weight status [4], levels of physical activity [5], tobacco smoking habits, and sleep habits [6].

Physical activity is associated with changes in gut microbial composition and an increase in butyrate-producing bacteria. It increases fecal butyrate concentrations in humans independent of diet, an essential mechanism for improving intestinal and cardiometabolic health with exercise [7]. Athletes are considered a specific population to study exercise’s chronic effects on the human body. Athletes’ nutrition follows specific recommendations, and each sport has characteristics that affect eating habits [8]. Therefore, it is difficult to establish the impact of different dietary patterns in studying the gut microbiota in elite athletes since sport has been shown to induce modifications to the gut microbiota per se [9]. For example, elite rugby players have been shown to have greater gut microbiota diversity than non-sporting controls. However, this difference is also attributable to a higher intake of whey protein as a supplement [10]. Higher-level martial arts athletes have an increased diversity and higher metabolic capacity of the gut microbiota than lower-level martial arts athletes [11]. Unlike rugby and martial arts, cycling [12] is a sport with higher endurance effort levels; transitioning from amateur to professional cycling induces increased levels of microorganisms such as Prevotella and Methanobrevibacter. Other endurance sports include the marathon [13] and cross-country skiing: these athletes have a reduced abundance of the principal genus of intestinal microbiota, Bacteroidetes, and a higher level of Prevotella. Compared to resistance training, it has been shown that endurance training is the metabolic process that involves the most significant changes in the composition of the gut microbiota through physical exercise [14]. In confirmation, subjects with higher cardiorespiratory fitness showed high diversity in their gut microbiota and a relative abundance of butyrate-producing bacteria, essential to gut microbiota homeostasis [15]. Therefore, the changes in the gut microbiota following physical exercise have yet to be definitively determined and fully understood but could depend on how the activity is practiced in terms of type, duration, intensity, and weekly frequency [16]. In addition, although the available evidence suggests that physical exercise alters the gut microbial composition, the metrics (e.g., abundance, evenness, richness, and diversity) have yet to be consistently reported [17]. Therefore, examining the microbiota in athletes fails to differentiate specific training regimens, as most situational sports involve mixed alternating aerobic and anaerobic training [18]. In particular, the gut microbiota of elite soccer players has yet to be reported in any study.

The present study hypothesized that the type, level, and quantity of exposure to exercise could influence the gut microbiota. To investigate the effects of levels of exercise on gut microbiota, the present study aimed to describe the differences and compare the gut microbiota characteristics of a group of elite male soccer players with a group of healthy sedentary men, a group of healthy males who practiced regular exercise and a group of healthy males who were exposed to a high amount of exercise.

## MATERIALS AND METHODS

### Participants recruitment

This cross-sectional study enrolled subjects with different physical activity habits during the previous two months to evaluate their gut microbiota composition differences. In particular, the inclusion criteria were: 1) male, 2) aged between 18 and 35 years, 3) normal weight condition, 4) absence of any metabolic disease, 5) nonsmokers, 6) white ethnicity, and 7) ≥ 9.5 points of adherence to the Mediterranean Diet (MedQ-Sus questionnaire) [19]. The exclusion criteria were: 1) treatment with antibiotics or probiotics during the previous two months, 2) acute gastrointestinal infections one month before the enrolment, and 3) chronic inflammatory bowel diseases (Crohn’s disease and ulcerative colitis).

Ninety-one subjects were enrolled for this study:

– 17 elite soccer players belonging to Italian Serie A (23.7 ± 4.2 yrs) with more than 6 hours of training per week, consisting of 5 sessions of about 90 minutes of mixed endurance and anaerobic exercise.
– 14 healthy men with high levels of physical training (24.5 ±5.6 yrs) with 6 hours of training per week, consisting of about three sessions of about 2 hours of mixed endurance and resistance exercise.
– 23 healthy men with moderate levels of physical training (29.3 ± 3.9 yrs) with up to 3 hours of training per week, consisting of about 3–4 sessions of about 30–45 minutes each of endurance exercise.
– 37 healthy men without exercise habits (28.1 ± 5.9 yrs).

All subjects provided written informed consent before beginning the study. The Anti-Doping Lab Institutional Review Board (Qatar), which conforms to the recommendations of the Declaration of Helsinki, approved the present study (IRB number: E201300004).

### Mediterranean Diet assessment

Before the start of the study, each subject underwent a nutritional assessment to assess their eating habits. Eligible participants were those who had adhered to a Mediterranean diet in the previous two months; those who did not follow these eating habits were not included in the study. Given the retrospective nature of the dietary assessment, the MedQ-Sus questionnaire [19] was chosen because this tool demonstrated both reliability in assessing adherence to the Mediterranean diet and the nutritional sustainability of food choices.

### Microbiome sample collection and DNA Extraction

Fecal samples were collected using the eNAT^®^ kit (608CS01R, COPAN, Italy), stored at room temperature, and delivered within five days to the laboratory (NEXT Genomics, Sesto Fiorentino, Italy) to perform the analysis. Total DNA was isolated using a Microbiome DNA Isolation Kit (Norgen Biotek Corp. Thorold, ON, Canada) from the microbial pellets following the manufacturer’s protocol. The quality and quantity of extracted DNA were assessed using a Qubit Fluorometer (Thermo Fisher Scientific, Waltham, Massachusetts, USA), and sample purity was confirmed spectrophotometrically by measuring the A260/A280 ratio.

### Sequencing and Bioinformatics Analysis

Amplicons of the variable V3–V4 region of the bacterial 16S rRNA gene, delimited through primers 341F and 805R, were sequenced in paired-end (2 × 250 cycles) on the Illumina MiSeq platform, according to the Illumina 16S Metagenomic Sequencing Library Preparation protocol. Raw sequences were processed with the DADA2 pipeline in R, which assigns taxonomy using the SILVA 138 database [20] as a reference with a 0.99 identity threshold. After pre-processing in R, the data were imported into QIIME 2 version 2021.4 for further analysis [21]. Sequence depth ranged from 12,334 to 435,143 with a mean of 232,129 ± 57,543 per sample.

### DNA Sequencing for Species Assignment

Species identified by NGS technology were then verified by sequencing 16S rRNA gene full-length amplicons (1550 bp) in end-point PCR using universal primer pair P0 (5’ GAAGAGTTTGATCCTGGCTCAG forward) and P6 (5’ CTACGGCTACCTTGTTACGA reverse). PCR was performed in 96-well detection plates in ABI prism 7000 (Applied Biosystems, Stockholm, Sweden) by amplifying the total DNA extracted from the same stool sample. The reaction was carried out using a Thermo Scientific PCR Master Mix (Thermo Fisher Scientific Inc., CA, USA) according to the manufacturer’s protocol. The amplicons obtained were sent to Eurofins Genomics (Eurofins Genomics Italy, Milano, Italy) for sequencing. Raw data were then analysed with BLASTN to verify the species assigned.

**TABLE 1:** Anthropometrics parameters and the MedQ-Sus questionnaire score in sedentary, moderately active, very active, and elite soccer players. Data are expressed as mean and standard deviation.

	No Physical Activity	Moderate Level of Physical Activity (< 3 hours × week)	High Level of Physical Activity (> 6 hours × week)	Elite Soccer Players
Height (cm)	175.7 ± 4.7	177.0 ± 6.7	179.8 ± 9.3	182.6 ± 6.7
Weight (kg)	69.4 ± 4.9	70.6 ± 6.3	73.7 ± 8.5	77.6 ± 6.9
BMI (kg/m^2^)	22.4 ± 1.0	22.5 ± 0.8	22.7 ± 0.8	23.2 ± 1.2
Cereals & cereal products	1.7 ± 0.5	1.5 ± 0.6	1.7 ± 0.6	1.5 ± 0.6
Legumes	1.3 ± 0.5	1.2 ± 0.9	1.5 ± 0.8	1.3 ± 0.8
Fresh vegetables	1.5 ± 0.6	1.2 ± 0.7	1.5 ± 0.6	1.5 ± 0.5
Fresh fruit	1.0 ± 0.9	1.5 ± 0.8	1.7 ± 0.5	1.5 ± 0.8
Dairy products	1.5 ± 0.8	1.5 ± 0.8	1.2 ± 0.7	1.3 ± 0.8
Fish and fish products	1.0 ± 0.6	1.3 ± 0.5	1.7 ± 0.5	1.3 ± 0.7
Meat and meat products	1.8 ± 0.4	1.5 ± 0.5	1.5 ± 0.5	1.5 ± 0.5
Olive oil	1.3 ± 0.5	1.2 ± 0.9	1.2 ± 0.7	1.5 ± 0.5
Total score	11.2 ± 1.5	10.8 ± 1.2	11.8 ± 1.6	11.5 ± 0.8

**Legend.** BMI: Body Mass Index.

### Statistical Analysis

All statistical analyses were completed with SPSSS (Version 18.0, SPSS Inc., IL, USA). Data are presented as mean±SD with significance at p ≤ 0.05. Descriptive statistics were computed on each variable, and the Shapiro-Wilk test was used to verify normality. Where statistical significance was shown, 95% confidence intervals (CI) were indicated to estimate the mean difference. Differences between populations were analyzed using a one-way ANOVA. Bonferroni’s post-hoc test was employed to identify any localized effects in the event of a significant difference. The standardized differences of effect size (ES, [95%CI]) in the selected variables were calculated. Threshold values for assessing magnitudes of the ES were > 0.20, 0.20, 0.60, 1.2, and 2.0 for trivial, small, moderate, large, and very large, respectively [22]. The z score was calculated to elaborate Figure 4.

### RESULTS

The anthropometric parameters and adherence to the Mediterranean Diet through the MedQ-Sus questionnaire, relating to the inclusion criteria of the different study populations, are presented in Table 1.

Descriptive statistics of the gut microbiota parameters of the different study populations are presented in Table 2.

**FIG. 1:**
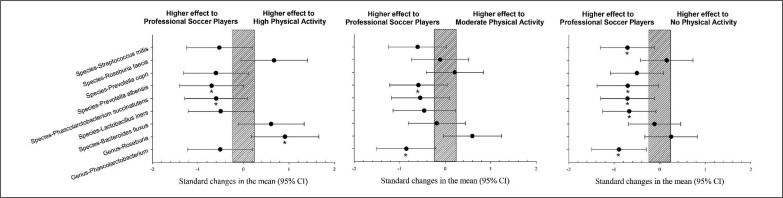
Differences in gut microbiota parameters between professional soccer players vs. population with high, moderate and no physical activity. CI: confidence interval.

Elite soccer players showed a significantly higher prevalence of Genus Phascolarctobacterium, species Lactobacillus iners, Phascolarctobacterium succinatutens, Prevotella albensis, and Streptococcus itis than the group without physical activity (p < 0.05). However, Genus Roseburia showed a significantly lower prevalence than the group with a high level of physical activity (Figure 1, p < 0.05).

**FIG. 2:**
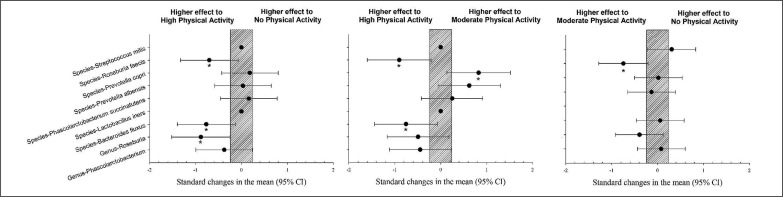
Differences in gut microbiota parameters between populations with high, moderate and no physical activity. CI: confidence interval.

The population with high physical activity showed a significantly higher prevalence of Bacteroides fluxus and Roseburia feces than those with moderate physical activity levels (p < 0.05). The population with a high level of physical activity showed a significantly higher prevalence of Roseburia feces, Bacteroides fluxus, and Genus Roseburia than the group with no physical activity (p < 0.05). The population with high physical activity showed a significantly lower prevalence of Prevotella copri compared to the group with a moderate level of physical activity (Figure 2, p < 0.05).

**FIG. 3:**
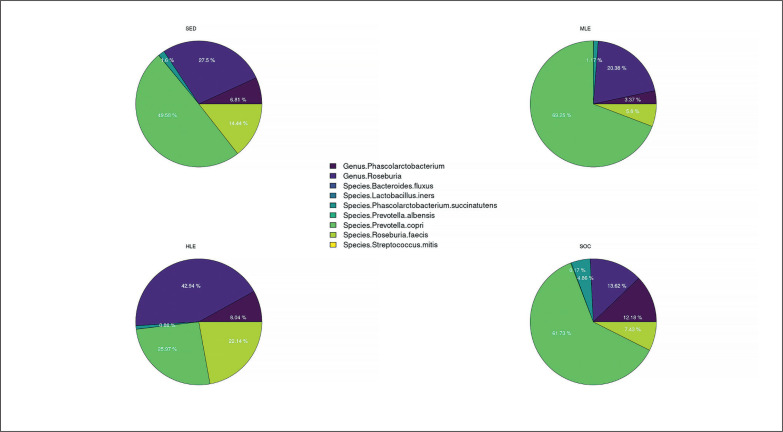
Percentage distribution of bacterial species. Furthermore, based on the prevalence of the various bacterial species in the different subject groups, the heatmap generated two clusters contrasting the bacterial species found in elite footballers and subjects with moderate physical activity vs. sedentary subjects and subjects with high physical activity.

**FIG. 4:**
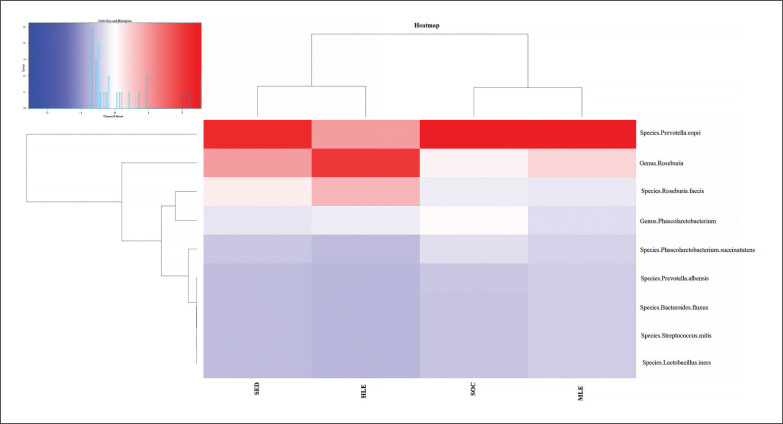
Distribution of bacterial species in the heatmap. The heatmap (constructed with R studio’s heatmap.2 function) depicts the distribution of the various bacterial species with different colors ranging from red to blue through intermediate colors. The red color represents a higher z score (>1.5), while the blue color represents a lower z score (<0.2).

The pie chart (Figure 3) and the heatmap (Figure 4) present the previously stated results. Each pie chart shows the percentage distribution of the most representative bacterial species within each group of subjects (SED (the group with no physical activity), MLE (moderate level of physical activity), HLE (high physical activity), SOC (elite soccer players)), the heatmap allows a comparison between the different populations in the different parameters analyzed. The results showed a significantly higher prevalence of Prevotella copri in subjects with moderate physical activity, followed by elite soccer players and, finally, sedentary subjects. In contrast, subjects with a high level of physical activity showed a significantly higher prevalence of Genus Roseburia and Species Roseburia feces than the other subject groups.

**TABLE 2:** Differences in gut microbiota parameters in sedentary, moderately active, very active and elite soccer players.

	No Physical Activity	Moderate Level of Physical Activity (< 3 hours ×week)	High Level of Physical Activity (> 6 hours × week)	Elite Soccer Players	ANOVA
Genus Phascolarctobacterium (%)	0.7390 ± 0.9401^a^	0.6666 ± 0.88242^a^	1.1295 ± 1.26701	2.1373 ± 2.51849	F 4.581 p 0.005

Genus Roseburia (%)	2.9828 ± 2.40412^b^	4.0301 ± 3.04254	6.0291 ± 5.36580	2.3905 ± 2.29816^b^	F 4.191 p 0.008

Species Bacteroides fluxus (%)	0.0009 ± 0.0018^b^	0.0008 ± 0.0017^b^	0.0028 ± 0.0038	0.0011 ± 0.0016	F 3.191 p 0.028

Species Lactobacillus iners (%)	0.0000 ± 0.0^a^	0.0000 ± 0.0	0.0000 ± 0.0	0.0001 ± 0.00027	F 2.936 p 0.038

Species Phascolarctobacterium succinatutens (%)	0.1735 ± 0.36710^a^	0.2307 ± 0.53093	0.1211 ± 0.19593	0.8535 ± 1.63961	F 3.377 p 0.022

Species Prevotella albensis (%)	0.0065 ± 0.01242^a^	0.0073 ± 0.00811^a^	0.0028 ± 0.00542^a^	0.0302 ± 0.05215	F 4.696 p 0.004

Species Prevotella copri (%)	5.3773 ± 9.47572^c^	13.6948 ± 13.51410	3.6473 ± 9.38986^c^	10.8339 ± 13.64028	F 3.563 p 0.017

Species Roseburia faecis (%)	1.5666 ± 1.58516b	1.1463 ± 0.92128b	3.1095 ± 3.36707	1.3045 ± 1.94235	F 3.472 p 0.019

Species Streptococcus mitis (%)	0.0000 ± 0.0000^a^	0.000 ± 0.000	0.000 ± 0.000	0.0002 ± 0.00051	F 3.008 p 0.035

F/B	1.37 ± 0.79	1.23 ± 0.60	1.61 ± 1.15	0.96 ± 0.62	F 1.946 p 0.127

**Legend.** F/B: Firmicutes to Bacteroidetes ratio.

a Differences with elite soccer players;

b Differences with high levels of physical activity;

c Differences with moderate levels of physical training.

## DISCUSSION

The gut microbiota comprises billions of bacteria, viruses, fungi, and protozoa. The present study showed nine gut microbiota parameters differences between the four sample groups studied. Five parameters were significantly greater in elite soccer players, three in the group with a high level of physical activity and one in the group with a moderate level of physical activity (Table 2, Figures 1 and 2). Some findings in the present study confirm earlier results, which describe how exercise appears to be associated with changes in gut microbial composition, an increase in butyrate-producing bacteria (e.g., Roseburia hominis, Faecalibacterium pausnitzii, and Ruminococcaceae) and increases in butyrate concentrations in faeces in rodent models [23] and in humans [24] regardless of diet.

In more detail, the results of our study about Genus Phascolarctobacterium and Species Phascolarctobacterium succinatutens are in line with a previous study [25] where higher levels of physical activity were associated with a greater abundance of this gut microbiota species. Phascolarctobacterium is a propionate producer, an SCFA that inhibits pro-inflammatory cascades by suppressing the activity of pro-inflammatory regulator nuclear factor kappa-B (NFκB) [26]. Our results showed that moderate physical activity is not enough to promote an increase in this gut microbiota species; a higher level of physical activity is probably needed.

The higher presence of Prevotella albensis and P. copri in soccer players also confirmed the results obtained in previous studies with cyclists and marathon runners compared to a sedentary population [12, 13].

In the present study, our results showed a gradual increase in Streptococcus mitis, Lactobacillus iners, and Roseburia species based on physical activity, excluding soccer players. The typology of competition and training in terms of high-intensity actions, type of specific/intermittent endurance, and muscle damage are probably responsible for this and are parameters for further study.

Bacteroides fluxus is a Gram-negative anaerobic bacillus associated with sarcopenia [27]. However, our results do not align with previous findings on the non-sporting adult population. The Firmicutes to Bacteroidetes ratio obtained from the present study is not in line with a previous study, which showed a direct relationship between this species and VO_2_ max [28]. While our soccer players’ values were comparable to those of healthy young adults in the Durk et al. study [28], although not significantly different, the sedentary subjects in our study showed higher F/B values.

The exercise-induced shifts in SCFA-producing taxa (Genus Phascolarctobacterium, species Prevotella copri, and Roseburia faecis) observed in this study corroborate data presented in rodent models [29] and in a previous cross-sectional study in humans relating fecal butyrate concentrations to aerobic physical performance and muscle turnover [30]. However, intense physical exercise probably induces a relatively low abundance of short-chain fatty acids (SCFA) and lactic acid-producing bacteria [30]

Previous studies have shown that BMI can influence gut microbiota compositions [7]. However, our study included only normal weight subjects; therefore, the differences could relate to the quantity of physical exercise.

The participants in this study, particularly the athletes, differed from numerous studies that have evaluated the gut microbiota in sports populations, as our sample followed the Mediterranean diet, which provided a lower protein intake and higher levels of fiber than the diets of the sportspeople in the previous studies [31]. Higher protein intake leads to differences in the composition of the gut microbiota because excessive protein ingestion leads to excess nitrogen substrates in the intestinal microbes, producing putrefactive fermentation products such as ammonia, hydrogen sulfide, amines, phenols, thiols, and indoles [32]. Conversely, low dietary fiber may decrease bowel movements and reduce gut microbiota diversity [32]. A variable that is difficult to control is the carbohydrate load, especially if the sample includes elite athletes. Carbohydrate load strictly depends on the physical training load required; in some cases, various carbohydrates can be used to maximize sports performance. However, our athletes preferred to eat resistant starch rather than monosaccharides. Proteins and carbohydrates are linked in intestinal digestion, as when the carbohydrate content decreases, putrefactive fermentation becomes more harmful [33]. It has been reported that high protein intake leads to DNA damage in the colon mucosa when the dietary intake of fermentable carbohydrates is low [34].

### Strength and limitations

This study has some strengths. This was the first study of the gut microbiota in elite soccer players. Secondly, the sample was homogeneous regarding body mass index, age, and eating habits. Therefore, the variable that differed between the four study groups in the sample was the level of physical activity. Furthermore, the sample size aligned with other studies investigating the human gut microbiota. Finally, subjects from the same territory were evaluated with the same conditions, place, and by the same operators.

However, limitations must be highlighted. The first limitation was the study’s cross-sectional design, with an inherent inability to generalize the cause-effect relationship. Secondly, according to the study design, the nutritional assessment was carried out retrospectively, therefore with an intrinsic limit to generalizability. However, a recently validated assessment method was used. Thirdly, although the total sample size is in line with other current studies, the sequencing method used, 16s RNA, may lose reliability in the subgroup analysis of the present study. Finally, this study did not analyze the biodiversity of the microbiota. However, this aspect was not in line with the aim of the study; furthermore, the sample size would not have allowed to obtain statistically significant results and consequently less reliable conclusions.

## CONCLUSIONS

The study of gut microbiota as related to the host’s health is a recent field of research, and there are currently numerous study directions. The relationship with physical activity, regardless of eating habits, is one of these new study directions. The results obtained from the present study, involving elite soccer players, expanded current knowledge and confirmed that increased levels of physical activity promote greater bioavailability of the gut microbiota. Our study described how exercise appears to be associated with changes in gut microbial composition, with an increase in butyrate-producing bacteria. To conclude, we have shown that high levels of physical activity positively contribute to health as they indirectly produce SCFA, which inhibits pro-inflammatory cascades.
